# Novel Insights into the Therapeutic Effect of Amentoflavone Against *Aeromonas hydrophila* Infection by Blocking the Activity of Aerolysin

**DOI:** 10.3390/ijms26052370

**Published:** 2025-03-06

**Authors:** Jing Dong, Shengping Li, Shun Zhou, Yongtao Liu, Qiuhong Yang, Ning Xu, Yibin Yang, Bo Cheng, Xiaohui Ai

**Affiliations:** 1 Yangtze River Fisheries Research Institute, Chinese Academy of Fishery Sciences, Wuhan 430223, China; 2 College of Fisheries and Life Science, Shanghai Ocean University, Shanghai 201306, China; 3 Key Laboratory of Aquatic Product Quality and Safety Control, Ministry of Agriculture and Rural Affairs, Chinese Academy of Fishery Sciences, Beijing 100141, China

**Keywords:** *Aeromonas hydrophila*, aerolysin, amentoflavone, molecular dynamics, anti-infective

## Abstract

*Aeromonas hydrophila* (*A. hydrophila*) is an opportunistic and foodborne pathogen widely spread in the environments, particularly aquatic environments. Diseases caused by *A. hydrophila* in freshwater aquaculture result in huge economic losses every year. The increasing emergence of antibiotic resistance has limited the application of antibiotics in aquaculture. Aerolysin (AerA), the main virulence factor produced by *A. hydrophila,* has been identified as a promising target for developing drugs controlling *A. hydrophila* infection. Here, we found that the natural compound amentoflavone (AMF) with the MIC of 512 μg/mL against *A. hydrophila* could dose-dependently reduce the hemolysis of AerA, ranging from 0.5 to 4 μg/mL. Molecular docking and dynamics simulation results predicted that AMF could directly bind to domain 3 of AerA via Pro333 and Trp375 residues. Then, the binding sites were confirmed by fluorescence quenching assays. The results of heptamer formation demonstrated that the binding of AMF could affect the formation of oligomers and result in the loss of pore-forming activity. Cell viability assay showed that AerA after treatment with AMF ranging from 0.5 to 4 μg/mL could significantly reduce AerA-mediated cytotoxicity. Moreover, experimental therapeutics results showed that channel catfish infected with *A. hydrophila* and then administered with 20 mg/kg AMF at intervals of 12 h for 3 days could increase the survival rate by 35% compared with the positive control after a 10-day observation. These findings provided a novel approach to developing anti-infective drugs and a promising candidate for controlling *A. hydrophila* infection in aquaculture.

## 1. Introduction

Food poisoning caused by pathogenic microorganisms has gained extensive attentions worldwide, and a large number of foodborne illnesses have occurred in various countries [1,2]. *A. hydrophila* is an opportunistic bacterium responsible for infections of fish, terrestrial animals, and humans. In freshwater aquaculture, *A. hydrophila* is an important bacterial pathogen that can cause motile aeromonad septicemia in a variety of fishes and bring huge economic losses to the industry [3]. In recent years, *A. hydrophila* has been recognized as a foodborne pathogen that can be isolated from not only sashimi, oysters, and salmon sushi but also meats, meat products, milk, and vegetables [4]. *A. hydrophila* isolated from food sources can lead to empyema and bacteremia of human with a high mortality rate and has become a severe problem to public health [1]. Antibiotics were used to deal with *A. hydrophila*-associated infections both in humans and in aquaculture. However, the occurrence of antibiotic resistance and residues in aquatic products limited the applications of antibiotics in aquaculture [5,6].

The pathogenicity and virulence of *A. hydrophila* are related to structure components, extracellular proteins, secretion systems, quorum sensing, and metal ions [7]. Aerolysin (AerA), a virulence indicator used to identify pathogenic *A. hydrophila*, is an essential virulence factor and can cause cell damage in a variety of mammalian cells [8]. AerA belongings to the pore-forming toxin (PFT) family; it is secreted as an inactive precursor and can be activated by cleaving the 43 residues in the C-terminus [9]. AerA can bind to N-glycosylated glycosylphosphatidylinositol (GPI)-anchored proteins on the surface of target cells and form heptamers with channel pores spanning the membrane of cells, which can cause the depletion of small ions, resulting in cell death [10]. A previous study demonstrated that the *A. hydrophila* strain deleting the AerA encoding gene significantly reduced the pathogenicity in a mice model [11]. These findings indicate that AerA plays a critical role in pathogenic *A. hydrophila* and can be developed as a promising target for identifying novel drugs against *A. hydrophila* infections based on the anti-virulence strategy.

**Figure 1:**
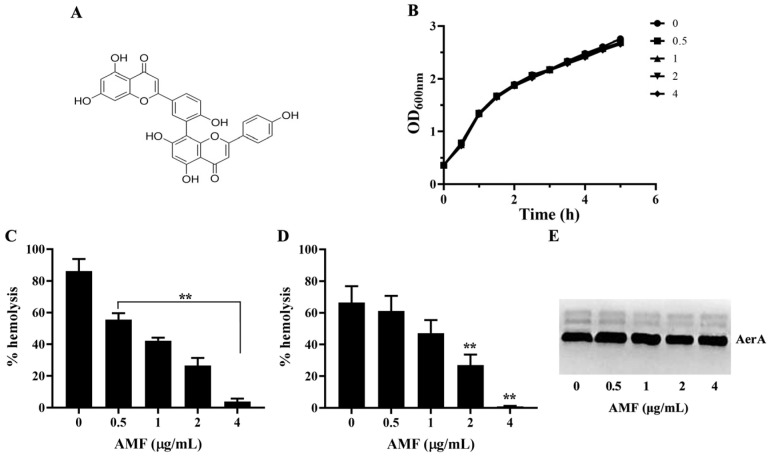
AMF without anti-*A. hydrophila* activity could directly inhibit the activity of AerA. (**A**) Chemical structure of AMF. (**B**) Growth curves of *A. hydrophila* co-cultured with various concentrations of AMF. (**C**) Hemolytic activities of bacterial supernatants co-cultured with AMF. (**D**) Hemolysis of purified AerA with indicated concentrations of AMF. (**E**) AerA production levels in bacterial supernatants after co-culturing with AMF. Data in (**C**,**D**) are mean ± SD of three independent experiments. ** *p* < 0.01 when compared with AMF-free group.

Amentoflavone (AMF, Figure 1A) is a biflavonoid compound firstly isolated from three plants of the *Selaginella* species by Okigawa et al. in 1971 [12]. In recent years, AMF could be isolated from more than 120 kinds of plants, most of which were used as traditional herbal medicine for thousands of years [13]. AMF has a number of biological activities, such as anti-tumor, anti-inflammatory, anti-oxidant, anti-virus, and anti-fungal activities [14,15,16,17,18]. However, there is little knowledge about the application of AMF in aquaculture. Here, we investigated the mechanism of AMF against *A. hydrophila* infection and provided a promising candidate for dealing with *A. hydrophila*-associated infections.

## 2. Results

### 2.1. AMF Had No Role on Bacterial Growth

The MICs were 512 µg/mL for AMF and 4 µg/mL for enrofloxacin. Moreover, the result of growth curves assay showed similar growth trend among the AMF-treated and AMF-free groups. These findings demonstrated that AMF could not affect the growth of *A. hydrophila* in 5 h at concentrations ranging from 0.5 to 4 μg/mL (Figure 1B).

### 2.2. AMF Decreased the Hemolytic Activity of AerA

As shown in Figure 1C, the hemolytic activities of bacterial supernatants obtained from bacterial suspensions co-cultured with AMF at concentrations ranging from 0.5 to 4 μg/mL were decreased to 55.54 ± 4.07, 42.35 ± 1.95, 26.63 ± 4.74, and 3.89 ± 1.92%, while 86.33 ± 7.43% of AMF-free group. When the concentration of AMF reached 0.5 μg/mL and above, statistical significance was observed. The results indicated that AMF could decrease hemolysis by affecting the activity or production of AerA. Therefore, hemolysis using purified AerA and Western blot assays was performed. As shown in Figure 1D, similar results were found when determining the hemolytic activities using purified AerA. After incubated with AMF at concentrations of 0.5, 1, 2, and 4 μg/mL, the hemolytic activities reduced to 61.15 ± 9.54, 47.32 ± 8.04, 27.07 ± 6.58, and 0.95 ± 0.37%, while 66.51 ± 10.30% of AMF-free group. AMF at concentrations higher than 1 μg/mL could remarkably reduce the hemolysis of purified AerA. The result of the Western blot showed that the addition of AMF in bacterial culture had no role in the production of AerA in bacterial supernatants (Figure 1E). Taken together, these results demonstrated that AMF could directly influence the activity of AerA at concentrations without anti-*A. hydrophila* activity.

### 2.3. Determination of Binding Sites

**Figure 2:**
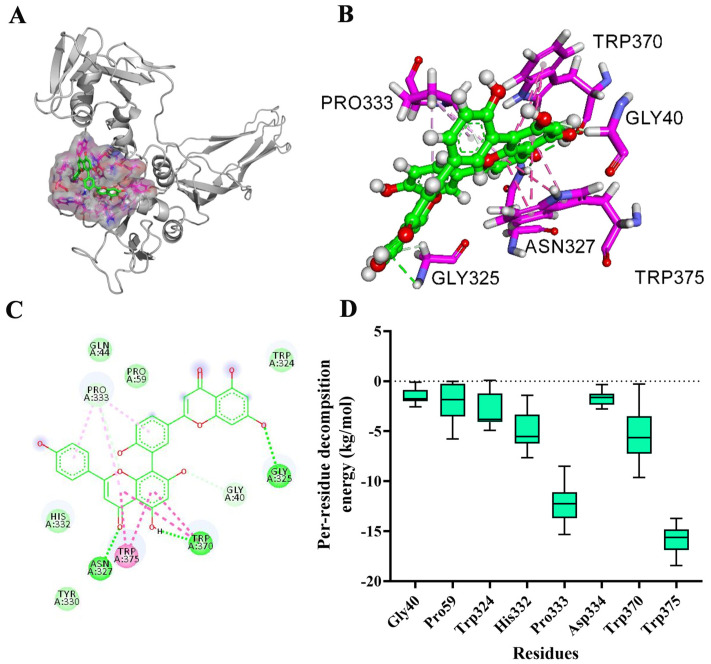
The predicted binding mode and potent binding sites of AMF-AerA complex. (**A**), Predicated binding mode of AMF with AerA. (**B**), Details of the binding mode with the lowest binding energy. (**C**), The interactions of AMF-AerA complex. (**D**), The decomposition of binding energy in the AMF-AerA complex is determined by the MM-GBSA method.

According to the result of the root-mean-square deviation (RMSD) values (Supplementary Figure S1), the system acquired equilibrium at the end of the simulation with an adequate force field and simulation protocol. The binding mode of AMF-AerA at a stable state is shown in Figure 2A and B. Gly40, Pro59, Trp324, His332, Pro333 Asp334, Trp370, and Trp375 were involved in the binding of AMF to AerA (Figure 2C). According to the results of the MM-GBSA method, the binding free energy of the AMF-AerA complex was calculated to be −22.16 kcal/mol. Pro333 and Trp375 were suggested to be the main binding sites of the complex according to the results of energy composition analysis (Figure 2D).

### 2.4. Confirmation of the Main Binding Sites of AMF-AerA Complex

Mutant proteins of Pro333A-AerA and Trp375A-AerA were purified to analyze the binding constants (*K_A_*) and the number of binding sites (n). As shown in Table 1, the *K_A_* of WT-AerA was 3.52 × 10^5^ L/mol, while 1.41 × 10^5^ L/mol and 1.38 × 10^5^ L/mol for Trp375A-AerA and Pro333A-AerA, respectively. The number of binding sites was closed to 1, indicating that AMF and AerA formed the complex at a mol ratio of 1:1. According to the results, the binding ability of WT-AerA, Trp375A-AerA, and Pro333A-AerA was decreased gradually, which matched the results of the MD simulation.

### 2.5. AMF Reduced Pore-Forming Activity of AerA

**Figure 3:**
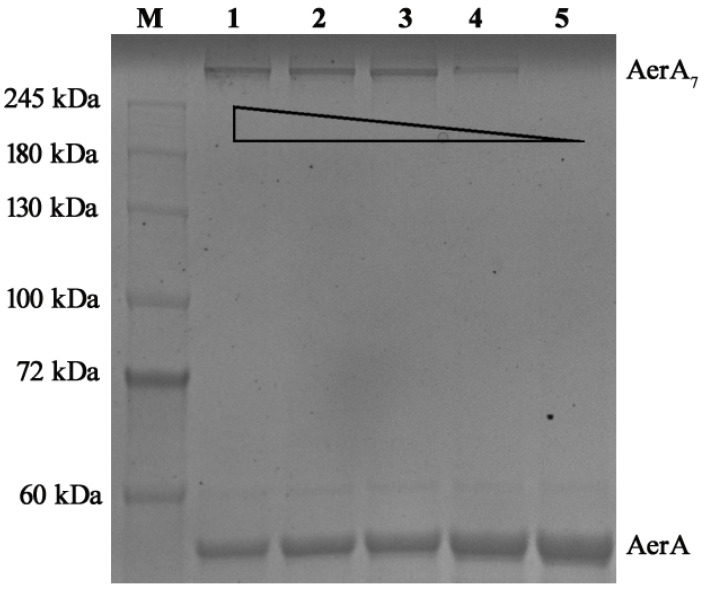
AMF blocked the oligomeric activity of AerA. M, protein molecular weight marker; Lane 1, WT-AerA; Lane 2, WT-AerA with AMF at mol ratio of 1:12.5; Lane 3, WT-AerA with AMF at mol ratio of 1:25; Lane 4, WT-AerA with AMF at mol ratio of 1:50; Lane 5, WT-AerA with AMF at mol ratio of 1:100. AerA represented the monomer of aerolysin, while AerA_7_ represented the heptamer of aerolysin.

As shown in Figure 3, AerA, after adding Hepes, could form heptamers at room temperature (RT), while AerA with indicated concentrations of AMF could reduce the production of heptamers in a dose-dependent manner. When the mol ratio of AerA and AMF reached 1:100, no heptamer was observed in the gel after staining (Figure 3). Taken together with the binding sites confirmation assay, these findings demonstrated that AMF could bind to Trp375 and Pro333 of WT-AerA, which disrupted the ability of AerA to form heptamers.

### 2.6. AMF Protected A549 Cells from AerA-Mediated Cell Injury

**Figure 4:**
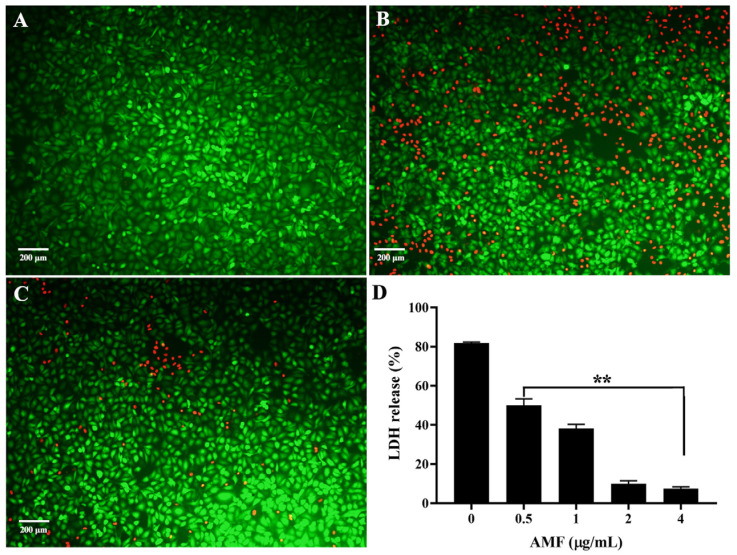
The protective effect of AMF on AerA-induced cell injury. (**A**) Untreated A549 cells; (**B**) Cells treated with AMF-free AerA; (**C**) Cells treated with AerA plus 4 μg/mL AMF; (**D**) LDH release of A549 cells in cell supernatants after treatment with AerA and indicated concentrations of AMF. Green indicates live cells, red indicates dead cells. Data in (**D**) are mean ± SD of three independent experiments. ** *p* < 0.01 when compared with AMF-free group.

As shown in Figure 4A, untreated cells were almost stained green by calcein-AM, representing the alive cells, while red fluorescence was observed in cells treated with AerA after staining with propidium iodide, indicating dead cells (Figure 4B). As shown in Figure 4C, cells after treatment with AerA plus AMF at a concentration of 4 μg/mL showed a visible decrease in red fluorescence, indicating that AMF treatment could increase the cell viability by neutralizing the cytotoxicity mediated by AerA. In addition, AMF could decrease the LDH release dose dependently, and statistical significance was observed when AMF concentrations reached 0.5 μg/mL and above compared with the AMF-free group (Figure 4D).

### 2.7. AMF Decreased the Mortality of Channel Catfish Infected with A. hydrophila

**Figure 5:**
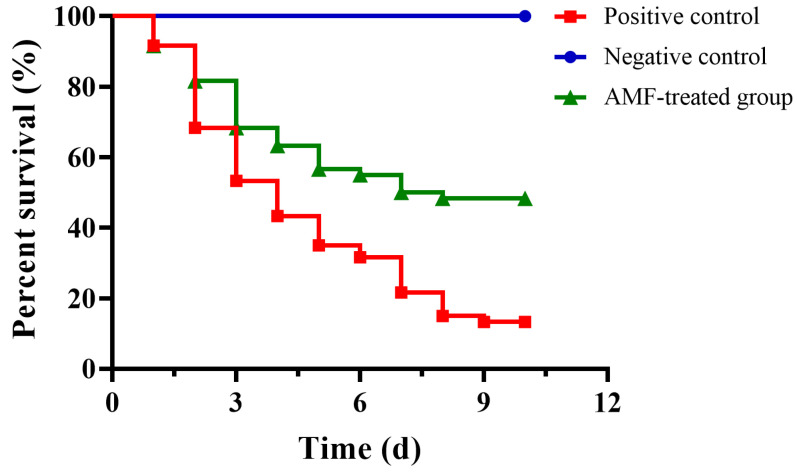
Experimental protective effect of AMF against *A. hydrophila* infection. Fish post *A. hydrophila* infection was given AMF at a dose of 20 mg/kg with intervals of 12 h for 3 days.

The above results demonstrated that AMF could reduce the virulence of *A. hydrophila* in vitro. As shown in Figure 5, deaths were observed 24 h post-infection in positive control and AMF-treated groups; the mortality of the positive control group was 86.67% at the end of the experiment, while the mortality of fish in the AMF-treated group was 51.67%. All fish in the negative control group were alive during the experimental period (Figure 5). The data were processed by Kaplan–Meier analysis and log-rank test; we found that fish administered 20 mg/kg AMF could significantly reduce the mortality caused by *A. hydrophila* infection compared with the positive control group.

## 3. Discussion

Multidrug-resistant *A. hydrophila* can be isolated from a wide variety of environmental and clinical sources, which is considered an emerging pathogen in humans, especially the elderly and children who are immunocompromised [19,20]. Similarly, *A. hydrophila* infections have caused huge economic losses in fish farming [21]. In the past, antibiotics were used as the primary drugs against pathogenic bacteria, while antibiotic-resistance genes were widely detected in *A. hydrophila* and indicated the failure of antibiotic therapy [22]. Previous studies demonstrated that AMF had significant antimicrobial activities against various bacteria, such as *Enterococcus faecium*, *Escherichia coli,* and *Pseudomonas aeruginosa,* with MICs ranging from 4 to 32 μg/mL [23]. Although previous studies demonstrated that AMF had several biological activities, we found little knowledge of its applications in aquaculture. As the main source of AMF, *Selaginella* species were reported to have no inhibitory activities against *A. hydrophila* and *A. veronii,* according to previous studies [24,25]. Here, we found that the MIC of AMF against *A. hydrophila* XS-91-4-1 was 512 μg/mL, much higher than the reported bacteria above, indicating that AMF had little anti- *A. hydrophila* activity. The MIC of enrofloxacin was 4 μg/mL, indicating that *A. hydrophila* XS-91-4-1 was resistant to enrofloxacin. Therefore, AMF would provide much less selective pressure to *A. hydrophila* at our experimental conditions compared with antibiotics.

Phytotherapy has been defined as a novel and promising alternative to antibiotic therapy, especially phytomedicines targeting the virulence factors of bacterial pathogens [26,27]. Phytomedicines inhibiting QS were well studied in *A. hydrophila* and followed by drugs that inhibit biofilms or other virulence factors [28,29]. Although various virulence factors were regulated by QS, the mechanism of AI-1 QS systems was only simply illuminated by constructing QS-related genes mutant strains [30,31]. Therefore, more labor intensity is needed to evaluate the effect of QS inhibitors on different virulence phenotypes and mechanisms. PFTs can be produced by both Gram-positive and Gram-negative bacteria, which are regarded as virulence factors involving bacterial dissemination and colonization [32]. Therefore, PFTs have been identified as targets for developing anti-infective drugs against bacterial infections, such as α-hemolysin (Hla) produced by *Staphylococcus aureus*, listeriolysin O (LLO) produced by *Listeria monocytogenes*, pneumolysin (PLY) produced by *Streptococcus pneumonia* and AerA produced by *A. hydrophila*. AerA belonging to β-PFTs is one of the most well-studied toxins and is a promising target for exploiting drugs against *A. hydrophila* infections [33]. Manikandan Arumugam et al. found that some compounds isolated from agricultural wastes, groundnut shells, and black gram pods had the potential ability to bind to AerA with hydrogen bonds after being analyzed by simulation dynamics; the findings provided a feasible solution for treating *A. hydrophila* infections [34]. However, the results obtained from computational methods were not verified by biological assays. In the present study, we identified AMF as the inhibitor of AerA by determining the hemolytic activity of AerA co-incubated with indicated concentrations of AMF. According to the results, we found that AMF at 2 μg/mL could statistically reduce the hemolysis mediated by purified AerA, while 0.5 μg/mL could remarkably decrease AerA-mediated hemolysis in bacterial supernatants. The concentration of AMF affecting hemolysis was much lower than its MIC. Furthermore, the mortality induced by *A. hydrophila* infection decreased by 35% after AMF treatment compared with the positive control group; the result indicated that AMF interfering with the pore-forming activity could reduce the pathogenicity of *A. hydrophila* to channel catfish rather than affecting the growth of the bacteria. The bioactivity of AMF, such as its anti-inflammatory effect, might help channel catfish in struggling with *A. hydrophila* infection, which needs to be further clarified.

Jamie Rossjohn et al. demonstrated that aerolysin was composed of 4 domains: Pro333 and Trp375 binding sites of AMF-AerA complex belonging to domain 3, which was responsible for receptor binding and oligomerization [35]. Trp375 was identified as the potent residue responsible for the binding to the receptor. However, the hemolytic activity assay using Pro333A-AerA and Trp375A-AerA showed similar hemolysis to WT-AerA (Supplementary Figure S2), indicating that Pro333 and Trp375 could not affect the binding ability of AerA to the receptor. The oligomerization assay demonstrated that AMF could disrupt the heptamer formation of AerA. Thus, it is reasonable to believe that Pro333 and Trp375 residues are involved in the oligomerization of AerA when co-incubated with AMF. Although previously reported AerA inhibitors luteolin and morin shared a similar chemical structure with AMF, the binding sites to AerA were absolutely different [36,37]. Previous studies have demonstrated that AMF could hinder the activities of LLO, PLY, suilysin produced by *Streptococcus suis*, α-toxin, and perfringolysin O secreted by *Clostridium perfringens* by disrupting the formation of oligomers [38,39,40,41]. Unfortunately, the main binding sites of AMF to the toxins mentioned above were not all determined, but PLY. Compared with the above studies, the inhibitory concentrations of AMF against LLO, PLY, suilysin, α-toxin, and perfringolysin O were higher than against AerA, suggesting that the binding energy of AMF-AerA complex was much higher than others. Moreover, the differences in inhibitory concentrations in vitro might affect the dose and effect of AMF in animal infection models. However, due to the huge difference in target animals and route of administration, the therapeutic effects of AMF against *A. hydrophila* infection could not be simply compared with previous studies. To our knowledge, this study was the first report determining the binding sites and mechanism of AMF-complex. AMF was first isolated from the plant *Selaginella*, which was widely used as an herbal medicine to treat several diseases in China [12,42]. The study provided a candidate compound for treating *A. hydrophila* infections in aquaculture and enriched the function of *Selaginella* in treating infectious diseases.

## 4. Materials and Methods

### 4.1. Microorganisms and Reagents

*A. hydrophila* strain XS-91-4-1 and recombinant AerA were stored in our laboratory. AMF was purchased from Sichuan Victory Biological Technology Co., Ltd. (Chengdu, China), while enrofloxacin was obtained from the National Institutes for Food and Drug Control (Beijing, China). AMF and enrofloxacin were prepared in DMSO for in vitro studies. For the in vivo study, AMF was dissolved in sterile PBS and adjusted the pH to about 9.0.

### 4.2. Minimum Inhibitory Concentrations (MICs) Determination

MICs of AMF and enrofloxacin against *A. hydrophila* were determined according to the guidance of CLSI [37]. Briefly, AMF and enrofloxacin were double diluted using MH medium to obtain final concentrations ranging from 1024 to 2 μg/mL for AMF, while 128 to 0.25 μg/mL for enrofloxacin in a 96-well plate. *A. hydrophila* XS-91-4-1 was cultured in LB medium at 28 °C to logarithmic phase, and bacterial cells were obtained by centrifugation. After washing, the cells were then standardized to a 0.5 McFarland in MH medium. 100 μL bacterial suspension at 5 × 10^5^ cfu/mL was added to each well, and the plate was further incubated at 28 °C for 16–18 h. The well containing the lowest concentration without bacterial growth was defined as the MIC of the strain.

### 4.3. Growth Curves

An overnight bacterial culture was sub-inoculated to 100 mL fresh LB medium and cultured at 28 °C to obtain an optical density (OD) of 0.3. The bacterial suspension was separated into 5 glass flasks at volumes of 10 mL, and then AMF was added to each flask to final concentrations of 0.5 to 4 μg/mL. Bacterial culture without AMF was used as a drug-free control group. The cultures were then incubated in a thermostatic shaker (150 rpm) for 5 h. The growth of bacteria with different concentrations of AMF was monitored by determining OD_600nm_ every 30 min using a spectrophotometer.

### 4.4. Hemolytic Assays

The hemolytic activity assay was performed using bacterial supernatants co-cultured with indicated concentrations of AMF and purified AerA. To investigate the hemolytic activity of bacterial supernatants, *A. hydrophila* XS-91-4-1 was cultured in the LB medium to obtain an OD_600nm_ of 0.3. The bacterial suspension was separated into glass flasks, and AMF was added to each flask to reach the final concentrations of 0.5, 1, 2, and 4 μg/mL. The mixtures were further cultured to an OD_600nm_ of 1.5, and bacterial supernatants were acquired by centrifugation. The hemolytic activity assay system was composed of 100 μL trypsin-activated supernatant, 25 µL freshly washed sheep erythrocytes, and 875 µL hemolysis buffer. The system was incubated at 37 °C for 15 min. For the activity of purified AerA, 5 μg/mL AerA in 975 μL hemolytic buffer was pre-incubated with indicated concentrations of AMF at 37 °C for 15 min, and then 25 µL sheep erythrocytes were added to the mixtures and further incubated at 37 °C for 15 min. Unlyzed red blood cells were removed by centrifugation, and the OD_543nm_ values of the supernatants were determined. A group of red blood cells treated with 0.1% Triton X-100 served as the positive control.

### 4.5. Immuno-Blot

Bacterial supernatants obtained for hemolytic activity assay were used for immune-blot assay. The total proteins in the supernatants were firstly determined by a Pierce^TM^ BCA protein assay kit (Thermo Fisher Scientific, Waltham, MA, USA). Then, the supernatants were sampled and loaded onto a sodium dodecyl sulfate (SDS)-polyacrylamide (12%) gel to separate the proteins. After electrophoresis, proteins in the gel were transferred to a PVDF membrane. Followed by blocking and incubating with a primary anti-AerA polyclonal antibody (prepared in our laboratory) and a goat anti-rabbit IgG conjugated with HRP (Dingguo, Beijing, China), the levels of AerA in the membrane were detected by enhanced chemiluminescence (ECL) detection regents (Yisheng, Shanghai, China).

### 4.6. Molecular Docking

The X-ray structure of AerA (PDB ID: 1PRE) and the chemical structure of AMF were obtained from the RCSB Protein Data Bank and PubChem database, respectively. AutoDock Vina 1.5.6 was used to carry out molecular docking. AerA was defined as a receptor, while AMF was a ligand. Firstly, polar hydrogen atoms and Gasteiger charges were added to AMF, and then non-polar hydrogen atoms were merged by AutoDock Tools software (ADT, version 1.5.6). A conformational search within the Box range (X: 112.458, Y: 125.164, Z: 178.117) was performed by the program. The docking results were scored by the conformation, orientation, position, and energy of the AMF-AerA complex, and proper conformation was then chosen for further molecular docking analysis.

### 4.7. Molecular Dynamics (MD) Simulation

The conformation of AMF-AerA complex acquired by molecular docking was then improved by MD simulation using GROMACS 19.6 package. TIP3P explicit water model was used to regulate the AMF-AerA complex, and a proper amount of sodium/chloride counterion was added to the system to obtain the neutralization of the system. Then, the amber99sb force field was performed to minimize the energy with the steepest descent method. After that, canonical ensemble simulation (NVT, 400 ps) and isothermal isobaric simulation (NPT, 400 ps) were used to guarantee that the system could endure constant temperature and pressure (300 K, 1 bar). Finally, a 50 ns MD simulation was performed, and the binding free energy of the complex was calculated using the MM-GBSA method.

### 4.8. Expression and Purification of AerA Mutants

Plasmids encoding P333A-pAerA and W375A-pAerA were constructed using a QuikChange XL site-directed mutagenesis kit (Agilent, Santa Clara, CA, USA) according to the instructions [36]. Primer pairs for generating the mutant plasmids are listed in Table 2. After sequencing, plasmids were transformed into BL21(DE3) competent cells. P333A-pAerA and W375A-pAerA were expressed by the addition of IPTG at a final concentration of 0.2 mM at 16 °C overnight. Proteins were purified by affinity chromatography and then activated by the addition of trypsin. Proteins were stored at −80 °C after being concentrated by an Amicon^®^ Pro affinity concentrator (Millipore, Billerica, MA, USA).

### 4.9. Fluorescence Quenching Assay

The fluorescence quenching assay was performed to determine the binding constants of the AMF-AerA complex, according to previous studies [43,44]. Briefly, the successively aliquot amount of AMF was added to a 3 mL solution of AerA or its mutants at the concentration of 1 μM. The excitation wavelength of the fluorescence spectrofluorimetry was set up to 280 nm with a 5 nm band-pass, while the emission wavelength was 345 nm with a 10 nm band-pass, respectively. The binding constants of AerA and its mutants with AMF were determined according to Stern-Volmer and Van’t Hoff equations.

### 4.10. Oligomerization Assay

The inhibitory effect of AMF against the formation of heptamer was determined using WT-AerA and AMF at mol ratios similar to hemolytic activity assay according to the method reported previously with some modification [45]. Briefly, WT-AerA was pre-incubated with AMF as performed in hemolytic assays at a whole volume of 20 μL for 15 min at 37 °C, 1 μL Hepes at the concentration of 1 M was then added to each sample to maintain the pH of oligomerization, and the mixtures were further incubated at RT for 1 h. All samples were analyzed after electrophoresis with 8% SDS-PAGE gel.

### 4.11. Cytotoxicity Assay

A549 cells were cultured in DMEM plus 10% fetal bovine serum at 37 °C with 5% CO_2_. Cells with a density of 1.5 × 10^5^ were seeded into a 96-well plate after being digested with trypsin and incubated overnight. AerA was pre-incubated with indicated concentrations of AMF for 15 min at 37 °C, and then the mixtures were added to each well. Cells were further incubated for 2 h. Cell supernatants were removed after centrifugation, while cells were washed twice with sterile PBS. Then, the live/dead regents with calcein-AM and propidium iodide were added to each well and incubated for 15 min at 37 °C. Live cells after treatment were stained green, while dead cells were stained red, respectively. Images were captured using a fluorescence microscope (Olympus, Tokyo, Japan). Cell supernatants were used for the LDH release assay according to the instructions supplied by the manufacturer.

### 4.12. Animal Study

The animal study was carried out to determine the protective effect of AMF against *A. hydrophila* infection. The study was performed with the approval of the Animal Welfare and Research Ethics Committee of the Yangtze River Fisheries Research Institute. One hundred eighty healthy channel catfish weighing 100 ± 10 g were used for establishing the infection model. Fish were divided into 3 groups; each group contained 3 biological repeats, and each repeat contained 20 fish. *A. hydrophila* XS-91-4-1 was cultured in LB medium to mid-logarithmic phase, and then bacterial cells were harvested by centrifugation. After being washed three times with sterile PBS, the density of bacterial cells was adjusted to about 1.5 × 10^9^ cfu/mL. Fish were firstly anesthetized by tricaine methanesulphonate (MS-222, 250 μg/mL). Then, 100 μL bacterial suspension was intraperitoneally injected into each fish in the AMF-treated group and positive control group, while fish in the negative control group were injected with 100 μL sterile PBS. Fish in the AMF-treated group were administered with 20 mg/kg AMF 6 h post-infection and then 12 h intervals for 3 days. Fish in positive and negative control groups were given sterile PBS (pH = 9.0) as control. Fish were maintained for 10 days post-infection, and deaths were recorded every day.

### 4.13. Statistical Analysis

Statistical analysis was performed using GraphPad Prism 8.0 software. Data were analyzed by Student’s *t*-test to determine the statistical significance. *p* < 0.05 indicates statistical significance. The survival rate was processed by Kaplan–Meier analysis and log-rank test.

## 5. Conclusions

In the present study, we found that the natural compound AMF could directly bind to AerA and result in the decrease of hemolytic activity at concentrations without interfering with the growth of *A. hydrophila*. After the dynamics simulation assay, the binding sites were confirmed to be Pro333 and Trp375. We found that the binding of AMF to AerA caused the decrease of heptamers, which is responsible for forming pores in the cell membrane. The study clarified the mechanism of herbal medicines in controlling bacterial diseases and provided a promising candidate for dealing with *A. hydrophila* infections.

## Figures and Tables

**Table 1:** Relationship of the values of binding energy (Δ*G_bind_*) and binding constants (*K_A_*).

	WT-AerA	AerA-W375A	AerA-P333 A
Δ*G_bind_* (kcal/mol)	−22.16	−15.99	−12.34
*K_A_* (1 × 10^5^) L/mol	3.52	1.41	1.38
n	1.3178	1.1988	1.1959

**Table 2:** Primer pairs used for the mutants.

Primer	Sequence
P333A-F	CTGGTATACCCATGCGGACAACCGC
P333A-R	GCGGTTGTCCGCATGGGTATACCAG
W375A-F	GTGGTGGGACTGGAACGCGACCATACAGCAGAAC
W375A-R	GTTCTGCTGTATGGTCGCGTTCCAGTCCCACCAC

## Data Availability

The data that support the findings of this study are available from the corresponding author upon reasonable request.

## Supplementary Materials

The supporting information can be downloaded at https://www.mdpi.com/article/10.3390/ijms26052370/s1.
