# Supplementary material for: Novel Insights into the Therapeutic Effect of Amentoflavone Against Aeromonas hydrophila Infection by Blocking the Activity of Aerolysin
Source: Int J Mol Sci. 2025 Mar 6;26(5):2370. doi: 10.3390/ijms26052370 (PMC11900166; doi:10.3390/ijms26052370)
Supplement: Supplementary file 1 [file ijms-26-02370-s001.zip › ijms-3492648-supplementary.pdf]

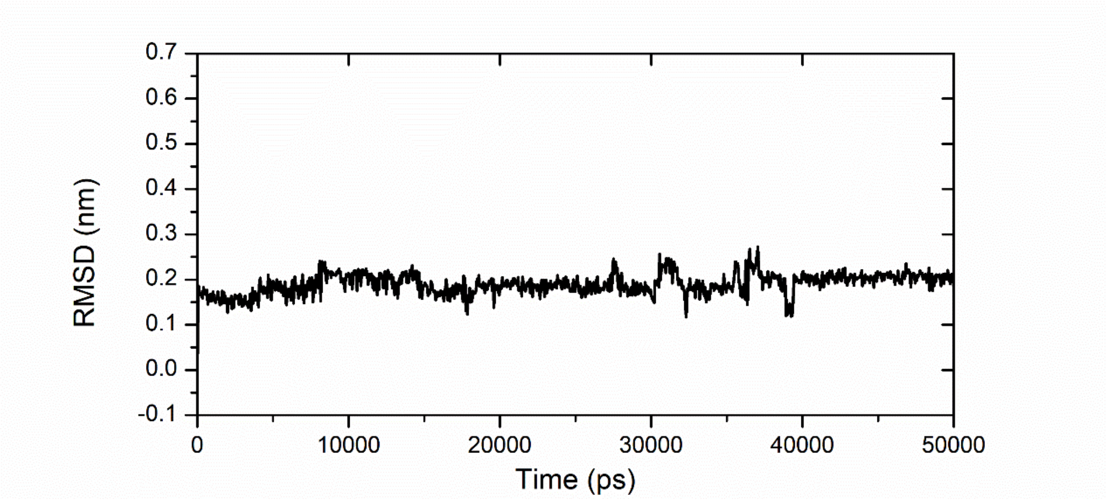

Supplementary Figure S1 The root means square deviation (RMSD) of the AerA-AMF complex

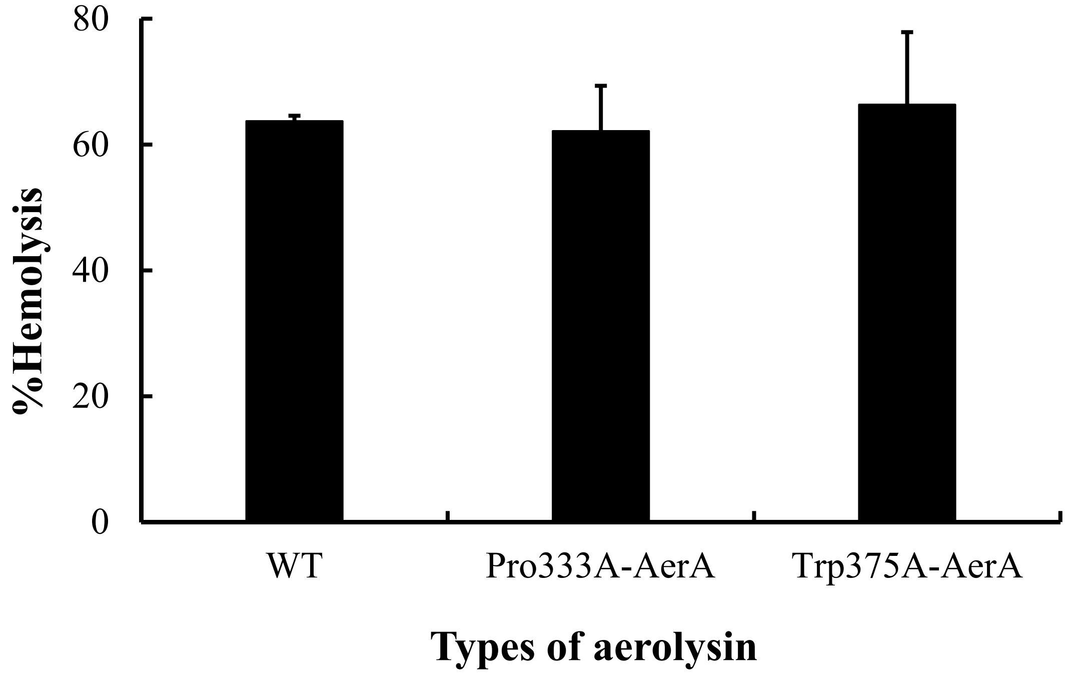

Supplementary Figure S2 Hemolytic activities of WT-AerA, Pro333A-AerA and Trp375A-AerA at the same concentration.
